# Prevalence and risk factors of metabolic-associated fatty liver disease in sub-Saharan Africa: a systematic review and meta-analysis

**DOI:** 10.3389/fgstr.2025.1506032

**Published:** 2025-04-01

**Authors:** Emmanuel M. Sindato, Violet Dismas Kajogoo, Gloria Ngajilo, Wondwossen Amogne Degu, Zahid Khan, Gideon Mlawa

**Affiliations:** ^1^ Division of Gastroenterology and Hepatology, Department of Internal Medicine, School of Medicine and Dentistry, The University of Dodoma, Dodoma, Tanzania; ^2^ Department of Clinical Trials, Tanzania Diabetes Association, Dar-es-salaam, Tanzania; ^3^ Division of Endocrinology, Department of Internal Medicine, Benjamin Mkapa Hospital, Dodoma, Tanzania; ^4^ Department of Internal Medicine, Addis Ababa University, Addis Ababa, Ethiopia; ^5^ Division of Cardiology, Department of Internal Medicine, Mid and South Essex NHS Foundation Trust, Southend-on-Sea, United Kingdom; ^6^ Division of Endocrinology and Diabetes Department of Internal Medicine, Barking, Havering and Redbridge University Hospitals National Health Service (NHS) Trust, London, United Kingdom

**Keywords:** metabolic-associated fatty liver disease, Sub-Saharan Africa, non-alcoholic fatty liver disease, metabolic associated steatohepatitis, prevalence, risk factors

## Abstract

**Background:**

Sub-Saharan Africa (SSA) is undergoing an epidemiological transition with a steady rise in non-communicable diseases. Among these diseases, metabolic dysfunction-associated fatty liver disease (MAFLD) has emerged as a rapidly increasing public health burden, but is inaccurately documented. We characterized the MAFLD prevalence and identified associated risk factors among adults in SSA.

**Methods:**

We searched PubMed/Medline, Cochrane, Embase, Web of Science, Google Scholar, and African Journals Online for studies looking into the prevalence of and the risk factors for MAFLD in SSA. Studies from 1990 in the English language were included, and the Preferred Reporting Items for Systematic Reviews and Meta-Analyses (PRISMA) guidelines were used for reporting. The quality of the studies was assessed using the Newcastle–Ottawa Scale. A random-effects model was used to estimate the prevalence and the risk factors with 95% confidence intervals (CIs). Meta-regression was used for the subgroup analyses to account for heterogeneity. Stata version 17 software was used for the analysis. This study was registered with PROSPERO (registration no. CRD42024506067).

**Results:**

A total of 538 studies were identified, with 22 included in the analysis. The overall prevalence of MAFLD was 29·21% (95%CI = 22.09–36.88, *p* < 0.05). Regionally, the results were: West, 34.36%; South, 26.92%; and East, 24.56%. The prevalence of MAFLD among people living with HIV was 13.02%, with diabetes was 37.06%, with hypertension was 36.75%, and with a body mass index above 25 kg/m^2^ was 46.05%. The prevalence was higher in women than in men (27.13% *vs*. 23.01%), as shown in studies conducted from 2000 onwards compared with those conducted between 2009 and 2019 (30.23% *vs*. 28.4%) and in studies with small sample sizes <500 than in studies with large sample sizes >500 (32.42% *vs*. 12.17%).

**Interpretation:**

MAFLD is highly prevalent in SSA, with a steady increasing magnitude. The significant risk factors included diabetes, hypertension, obesity, and female sex. This study underscores the emerging need of clinicians in SSA to screen MAFLD among patients at high risk and to instigate tailored care.

**Systematic review registration:**

https://www.crd.york.ac.uk/prospero/, identifier CRD42024506067.

## Introduction

Metabolic dysfunction-associated fatty liver disease (MAFLD) is a common type of chronic liver disease characterized by cardiometabolic risk factors and the hepatic steatosis affecting up to 30% of the population (1–7), with the highest burden in South America and the Middle East, followed by Asia, the USA, and Europe. In Europe and America, it is the second cause of end-stage liver disease, including liver cirrhosis and hepatocellular carcinoma (HCC), and the second cause of liver transplantation (8–10). Epidemiological studies have shown that Africa has the lowest burden of MAFLD, with a prevalence ranging from 10% to 28% (3). This proportion is projected to rise, as the African region is undergoing epidemiological transition with a particular steady rise in non-communicable diseases. Type 2 diabetes mellitus (DM), metabolic syndrome, and hypertension (HTN) are important risk factors for MAFLD (11–13).

MAFLD, also known as metabolic dysfunction-associated steatotic liver disease (MASLD), is the new nomenclature of the previous classification of nonalcoholic fatty liver disease (NAFLD). The new nomenclature was proposed around 2020 and was endorsed in 2023 by over 70 major liver societies and organizations, including the Society on Liver Disease in Africa (SOLDA), the American Association for the Study of Liver Diseases (AASLD), the European Association for the Study of the Liver (EASL), the American Gastroenterology Associates (AGA), and the Asian Pacific Association for the Study of the Liver (APASL), among others, following the multi-society Delphi consensus statement on the new fatty liver disease nomenclature (14–17). The new nomenclature addressed the stigma associated with the use of the word “alcohol” in the NAFLD classification. The new nomenclature uses a simplified criterion for the diagnosis based on simple and practical parameters. The new criteria require the presence of steatohepatitis plus any one of the following five cardiometabolic risks: body mass index (BMI) ≥25 kg/m^2^ or waist circumference (WC) >94 cm; fasting blood glucose (FBG) ≥5.6 mmol/L or HbA1c >5.7% or type 2 diabetes; blood pressure (BP) ≥130/85 mmHg or anti-hypertensive drug treatment; and plasma triglycerides ≥1.70 mmol/L, plasma high-density lipoprotein (HDL) cholesterol ≤1, or lipid-lowering treatment (14, 16). Furthermore, the overlap of steatohepatitis with alcohol consumption was addressed, in which a new terminology, “metabolic and alcohol-related/associated liver disease (MetALD),” evolved for moderate drinkers with cardiometabolic risks. Under this regard, moderate drinkers are classified based on the amount of alcohol consumed per week, as follows: 140–350 g/week for women and 210–420 g/week for men (14–17). Taking the new nomenclature into context, studies have shown that it has a comparable prevalence with NAFLD, but has a better identification pattern for patients with high cardiometabolic risks, improved patient awareness, and hepatic fibrosis diagnosis (15, 17, 18).

In the majority of patients, MAFLD is asymptomatic; however, some patients may present with a broad array of nonspecific symptoms, including exhaustion, malaise, and right upper abdominal discomfort (19, 20). Most patients will seek medical attention following an incidental hepatic steatosis finding on abdominal imaging and elevation of aminotransferase levels. Elevated transaminase levels in a patient with cardiometabolic risk should raise clinical suspicion for MALFD, although a normal transaminase level does not exclude MAFLD. Patients with MAFLD may have mild or moderate elevations of aspartate aminotransferase (AST) and alanine aminotransferase (ALT). It is important to note that the extent of transaminase elevation does not reflect the degree of hepatic inflammation or fibrosis (21). Imaging findings in patients with MAFLD include increased echogenicity on ultrasound, decreased hepatic attenuation on computed tomography (CT), and an increased fat signal on magnetic resonance imaging (MRI) (22). The disease spectrum of MAFLD ranges in severity from steatosis (hepatic fat accumulation without inflammation), steatohepatitis (hepatic fat accumulation with inflammation and hepatic injury), hepatic fibrosis, liver cirrhosis, and, consequently, HCC (1, 16, 21, 23).

The SSA region is undergoing epidemiological transition with the steady increase in the rates of cardiometabolic diseases, which are strongly associated with the increased prevalence of MAFLD, which could lead to complications such as end-stage liver disease primarily, liver cirrhosis, and HCC. However, to date, no systematic reviews of the prevalence of and the risk factors for MAFLD in the region have been published. One meta-analysis on chronic liver disease in Ethiopia, with a particular focus on the etiological spectra, was published in 2021. Therefore, this study aimed to characterize the burden by looking at the prevalence and the risk factors associated with MAFLD in SSA.

## Methods

### Search strategy

In this systematic review and meta-analysis, the Preferred Reporting Items for Systematic Reviews and Meta-Analyses (PRISMA) guidelines were used, and studies that reported on the prevalence of and risk factors for MAFLD in SSA in the English language were included. This study was registered with PROSPERO, with registration number CRD42024506067.

Electronic databases such as PubMed/Medline, Cochrane, Embase, Web of Science, and African Journals Online (AJOL) were used to search and retrieve articles published in English from 1990 to February 2024 using the following key terms: “Prevalence, Risk Factors, Nonalcoholic fatty liver disease, Metabolic dysfunction-associated fatty liver disease, metabolic dysfunction-associated steatotic liver disease, “metabolic and alcohol related/associated liver disease and SSA.” For PubMed, the Medical Subject Headings (MeSH) terms were used based on the research question. Boolean operators were also utilized to fine-tune the search terms. Furthermore, the authors manually searched the reference lists of the included studies and relevant review articles.

### Selection criteria

Eligibility was formulated using the PICOS (participants, interventions, comparisons, outcomes, study design) format, as detailed in **Table 1**.

**Table 1:** Eligibility criteria.

Criteria	Inclusion	Exclusion
Population	Patients aged 18 years with a diagnosis of MAFLD/NAFLD/MASLD/MASH	Not reporting MAFLD in SSA
		Studies in the pediatric population (aged less than 18 years)
		Studies reporting on MAFLD prevalence following autopsy
Intervention	MAFLD diagnostic modalities (abdominal USS: ultrasound scanning, CT, MRI, biopsy, fibro scan, and liver enzymes)	Reporting on other liver diseases (CHB and HCV), autoimmune hepatitis, hemochromatosis, or Wilson’s disease
Comparison	Comparing prevalence outcomes in different subgroups	Studies on age and time at acquisition of MAFLD infection and its effect
Outcome	Prevalence of and risk factors for MAFLD in SSA	Prevalence of and risk factors for MAFLD outside Africa
Study design	Cohort, case–control, and cross-sectional studies	Case reports, studies that are retrievable and not in the English language

Studies that did not report screening for excess alcohol consumption were interpreted with caution.

*MAFLD*, metabolic dysfunction-associated fatty liver disease; *NAFLD*, non-alcohol-associated fatty liver disease; *MASLD*, metabolic dysfunction-associated steatotic liver disease; *MASH*, metabolic dysfunction-associated steatohepatitis; *SSA*, Sub-Saharan Africa; *CHB*, chronic hepatitis B; *HCV*, hepatitis C virus.

MAFLD was defined as steatosis plus any one of the following: BMI ≥25 kg/m^2^ or WC >94 cm; fasting serum glucose ≥5.6 mmol/L or HbA1c >5.7% or type 2 diabetes; blood pressure ≥130/85 mmHg or anti-hypertensive drug treatment; and plasma triglycerides ≥1.70 mmol/L, plasma HDL cholesterol ≤1, or lipid-lowering treatment. MetSLD was defined using the MASLD criteria plus moderate or greater alcohol consumption.

The articles were imported from electronic databases into the Mendeley Reference Manager. Duplicates were removed and the titles, abstracts, and full texts were screened. Two authors performed the screening independently, and discrepancies between authors was discussed with a third reviewer and resolved by consensus.

### Data extraction, quality assessment, synthesis, and analysis

Two reviewers independently extracted data from the included studies using a standardized Excel data extraction form developed for the study. Discrepancies were resolved through discussion. The extracted data included author names, article publication year, country, study design, sample size, prevalence, and risk factors (e.g., categorical BMI, DM, HTN, and dyslipidemia) for MAFLD/NAFLD/MASLD in SSA.

The quality of the included studies was assessed using the Newcastle–Ottawa Scale for observational studies. Each study was graded as having a low, high, or unclear risk of bias for each domain and was color coded.

A random-effects model was used to estimate the pooled prevalence and the risk factors with 95% confidence intervals (CIs) from the studies. Heterogeneity between studies was estimated using Cochran’s *Q* statistic (*p* < 0.05 indicates low heterogeneity) and the *I* ^2^ statistic (<50% indicates low heterogeneity). Subgroup analyses to account for heterogeneity were performed, which examined associations between sex, BMI, study period, sample size, and region. A pooled prevalence was also determined among people living with HIV (PLHIV) and those who are diabetic and hypertensive. Stata version 17 software package was used for the analysis.

## Results

**Figure 1:**
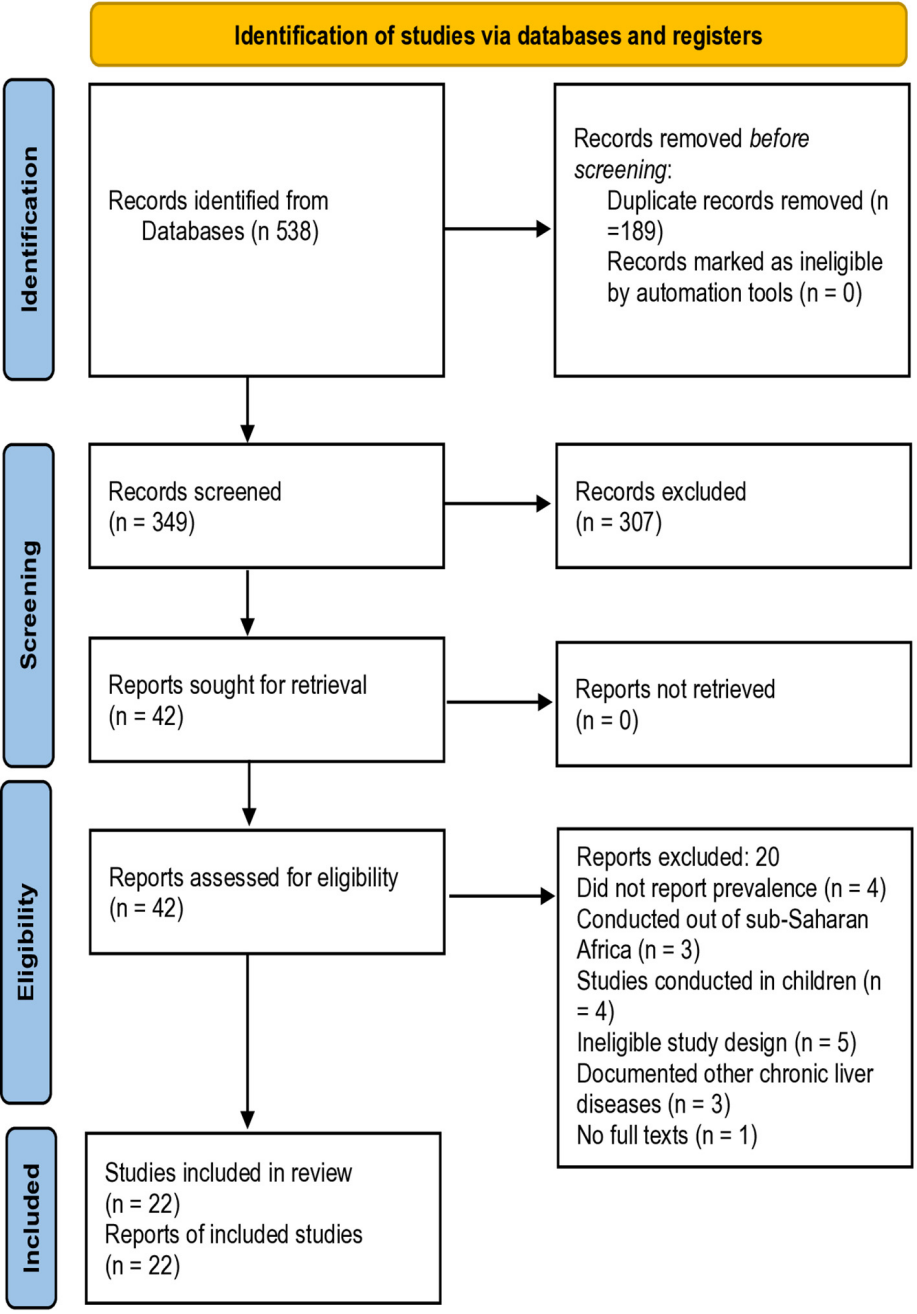
Preferred Reporting Items for Systematic Reviews and Meta-Analyses (PRISMA) flow diagram.

A total of 538 studies were identified. After removal of the duplicates, 349 records were retained for screening. The titles and abstracts were then screened, which excluded 307 records. The full texts of the remaining 42 records were then assessed for eligibility, from which 20 records were excluded. In the end, a total of 22 articles from 10 countries—Nigeria = 5, Sudan = 4, Ghana = 4, South Africa = 2, Kenya = 2, Ethiopia = 1, Zambia = 1, Côte d’Ivoire = 1, Uganda = 1, and Tanzania = 1—were included in the meta-analysis (**Figure 1**). Data from all the 22 (24–45) studies were included for the overall prevalence of NAFLD, 11 studies for the subgroup prevalence in patients with DM, 7 studies for HTN cases, 11 studies for sex, 9 studies for the risk factor BMI, and 3 studies for prevalence in people living with HIV (**Supplementary Table S1**).

### Quality assessment

The quality of the studies assessed according to the Newcastle–Ottawa Quality Assessment Scale (46) is shown in **Supplementary Table S1**. Overall, one study had moderate quality (Afolabi et al.), while the other studies had high quality.

The overall prevalence analysis included all of the 22 studies, with a total sample size of 5,813 individuals. The sample size range was from 80 to 1,463 individuals, within a study period from 2009 to 2024. The characteristics of the studies are shown in **Supplementary Table S2**.

**Figure 2:**
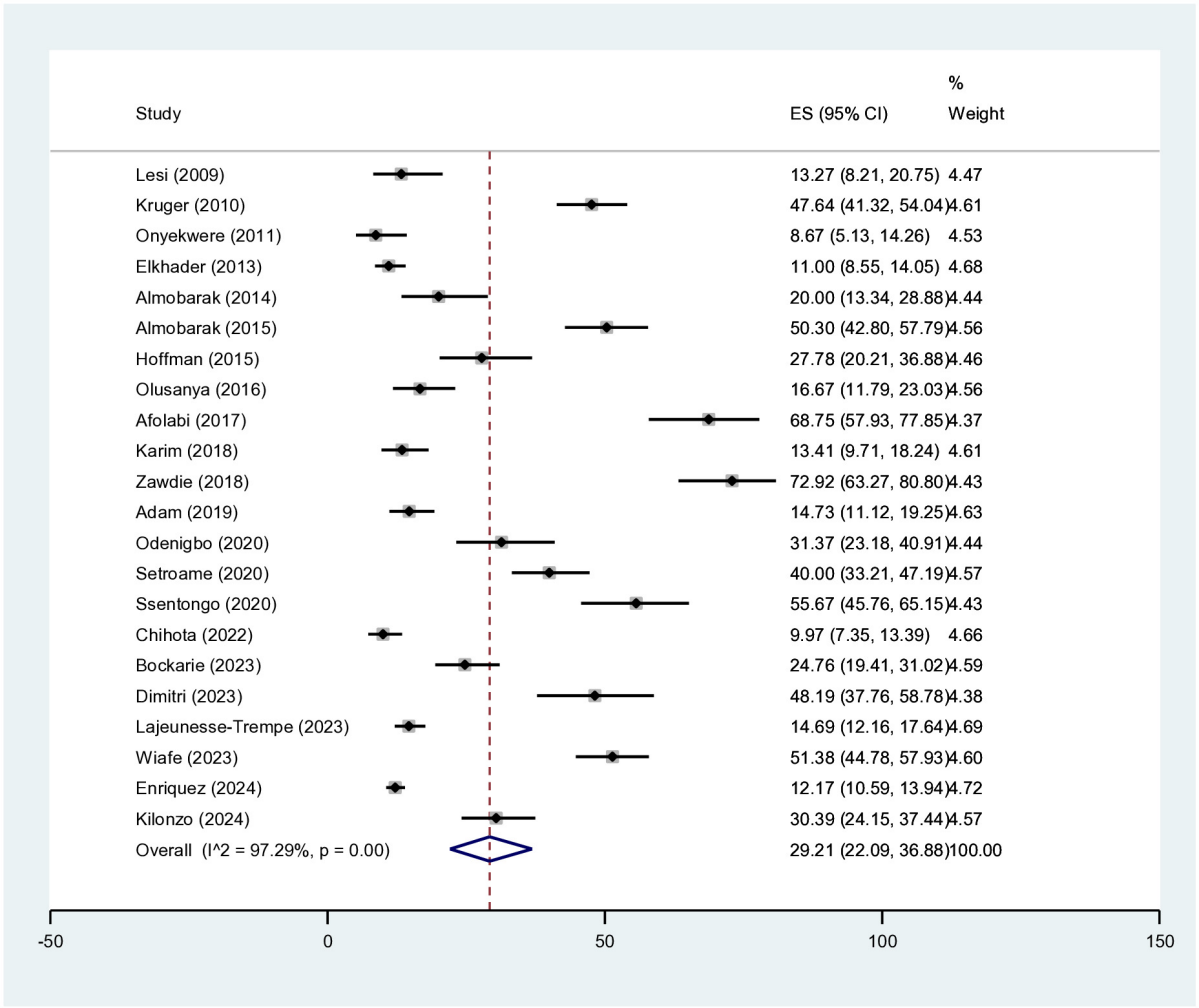
Overall metabolic-associated fatty liver disease (MAFLD) prevalence.

The overall prevalence of MAFLD was 29.21% (95%CI = 22.09–36.88). The study by Afolabi et al. had the highest prevalence (68.75%), while that by Onyekwere et al. had the lowest prevalence (8.67%). Both studies were conducted in Nigeria (**Figure 2**).

The regional prevalence was as follows: West, 34.36% (95%CI = 22.58–47.18), with a total of 10 studies and 1,406 participants; South, 26.92% (95%CI = 6.2–55.22), with a total of three studies and 722 participants; and East, 24.56% (95%CI = 16.08–34.16), with a total of nine studies and 3,685 participants (**Table 2**).

**Table 2:** Summary of the subgroup analyses.

Variable	No. of studies	Sample size	Prevalence% (95%CI)	Heterogeneity (%)	*p*-value
Region	10	1,406	34.36 (22.58–47.18)	95.88	<0.01
West					
South	3	722	26.92 (6.2–55.22)	0	<0.01
East	9	3,685	24.56 (16.08–34.16)	97.26	<0.01
Other comorbidities					
HIV	3	375	13.02 (2.19–30.45)	94.28	<0.01
Diabetes	11	1,066	37.06 (23.8–51.36)	95.31	<0.01
Hypertension	7	569	36.75 (25.86–48.33)	85.64	<0.01
BMI >25 kg/m^2^	9	999	46.05 (30.12–62.38)	96.03	<0.01
Sex					
Men	11	1,209	23.01 (14.77–32.4)	91.59	<0.01
Women	11	2,208	27.13 (16.91–38.71)	96.34	<0.01
Study period					
2020 onwards	10	3,560	30.23 (20.49–40.95)	97.43	<0.01
2009–2019	12	2,253	28.40 (17.52–40.7)	97.33	<0.01
Sample size					
<500	19	3,210	32.42 (23.78–41.7)	96.67	<0.01
>500	3	2,603	12.58 (10.77–14.52)	47.78	0.15

*CI*, confidence interval; *HIV*, human immunodeficiency virus; *BMI*, body mass index.

The prevalence of MAFLD among people with HIV was 13.02% (95%CI = 2.19–30.45), with a total of three studies and a sample size of 373 participants (**Table 2**). In diabetics, this was 37.06% (95%CI = 23.8–51.36), with a total of 11 studies and 1,066 participants (**Table 2**). The prevalence in hypertensives was 36.75% (95%CI = 25.86–48.33), with a total of seven studies and 569 participants (**Table 2**), while that in people with a BMI above 25 kg/m^2^ was 46.05% (95%CI = 30.12–62.38), with a total of nine studies and 999 participants (**Table 2**).

The prevalence of MAFLD was higher in females than males, 27.13% (95% CI 16.91 – 38.71) (**Table 2**) vs 23.01% (95% CI 14.77 – 32.4) (**Table 2**), higher in studies conducted from 2000+ as compared to those conducted between 2009-2019, 30.23% (95% CI 20.49 – 40.95) (**Table 2**) vs 28.4% (95% CI 17.52 – 40.7) (**Table 2**), and higher in studies with small sample sizes ≤500 than in studies with large sample sizes >500; 32.42% (23.78 – 41.7) (**Table 2**) vs 12.17% (10.58 – 13.14) (**Table 2**).

Considerable heterogeneity was observed overall between the prevalence studies and the subgroup analyses.

**Figure 3:**
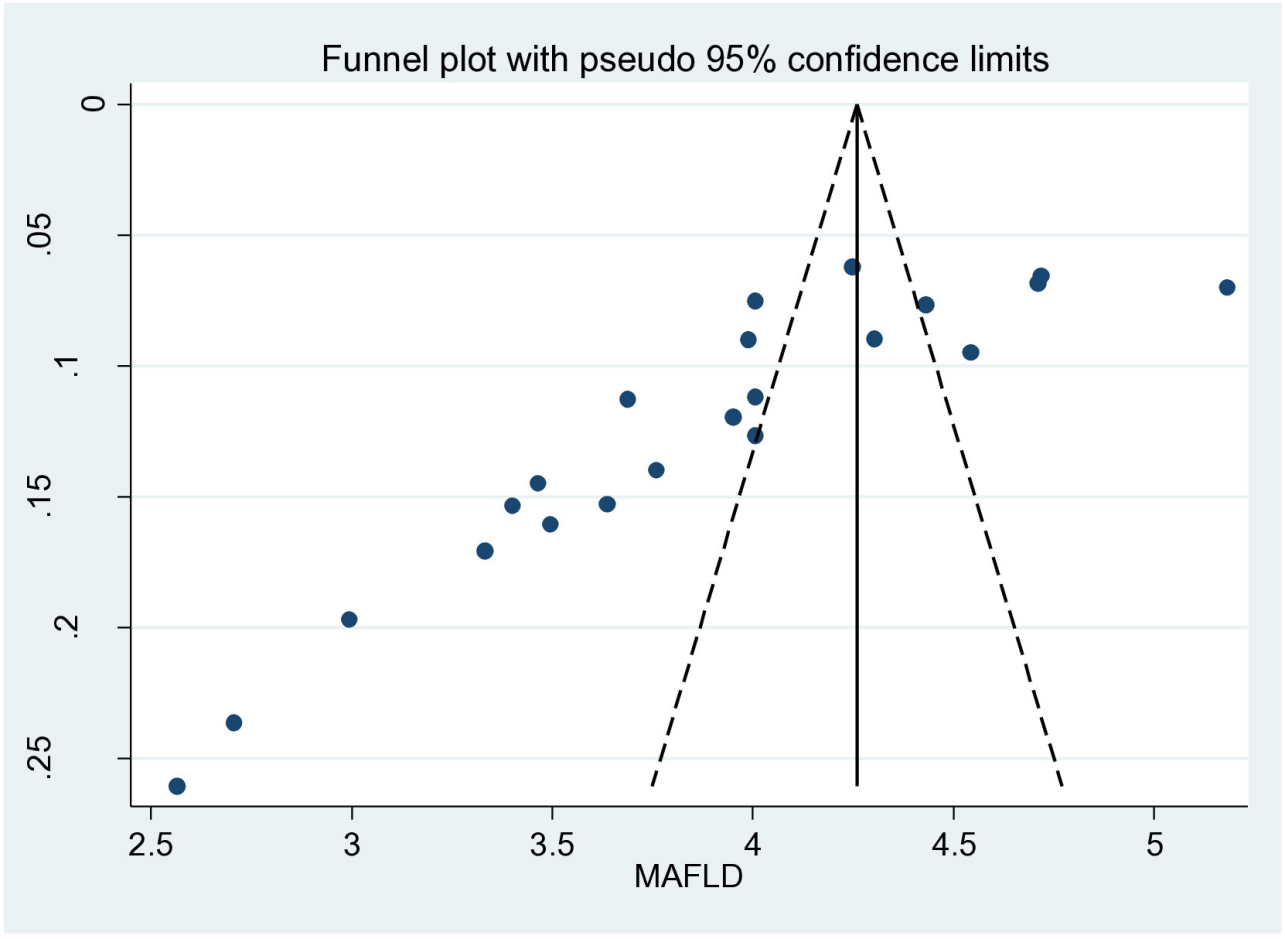
Funnel plot of the overall metabolic dysfunction-associated fatty liver disease (MAFLD) studies showing publication bias.

There was also an unequal scatter of the funnel plot, which could be due to small study bias or publication bias (**Figure 3**).

## Discussion

This review determined the prevalence of MAFLD in SSA, which was found to be 29.21%. Regionally, there was a higher prevalence in the West (34.36%) compared with the South (26.92%) and the East (24.54%). This was slightly more than the global estimates, where it was estimated to affect approximately 25% of the world’s population (47), but lower than the prevalence in Iran (33%) as of 2024 (48). The overall prevalence of MAFLD in the region was challenging to assess because, in February 2020, consensus by an international panel recommended the adoption of MAFLD as a more appropriate term; however, perspectives as to whether or not these changes are needed can vary substantially between different regions and healthcare systems (49). Nevertheless, the increase might be ascribed to the increased availability of healthcare and the concomitant rise in accurate NAFLD diagnosis, as well as to changes in lifestyle (50).

There were considerable variations in ethnicities, lifestyles, economic conditions, and disease epidemiology, which might have contributed to the wide variation in the prevalence of NAFLD (47). In a study conducted in different ethnic groups of South African women, the prevalence of metabolic syndrome was highest in the Indian subjects, being significantly higher than that observed in the Caucasian cohort, while the total energy expenditure was significantly higher in the African subjects compared with the Indian and Caucasian subjects (51). In a study done in the United States, the NAFLD prevalence was highest in Hispanic people, intermediate in Caucasian people, and lowest in African American people, although the differences between groups were smaller in the high-risk cohorts (range, 47.6%–55.5%) than in the population-based cohorts (range, 13.0%–22.9%) (52).

Higher prevalence rates were found in people with diabetes (37.06%), HTN (36.75%), and BMI >25 kg/m^2^ (46.05%), these being risk factors. Central obesity, a higher blood pressure, and a raised FBG were the predominant components that contributed to the syndrome in Ghanaian women (53). There was also a high prevalence of liver function test abnormalities in the group of patients with type 2 diabetes, and particularly so in the morbidly obese subjects in South Africa (54). A meta-analysis conducted in Mainland China had similar results, where there was a higher prevalence than the overall in those with diabetes (51.83% *vs*. 30.76% in non-diabetics) and in obese participants (66.21% *vs*. 11.72% in lean) (55). Similarly, a meta-analysis conducted in Latin America found type 2 DM or obesity to have a higher mean prevalence that reached 68% (56), as well as in a global epidemiology meta-analysis where the metabolic comorbidities associated with NAFLD included obesity (51.34%, 95%CI = 41.38–61.20), type 2 diabetes (22.51%, 95%CI = 17.92–27.89), hyperlipidemia (69.16%, 95%CI = 49.91–83.46), HTN (39.34%, 95%CI = 33.15–45.88), and metabolic syndrome (42.54%, 95%CI = 30.06–56.05) (1).

Our meta-analysis found a higher prevalence in women (27.13%) than in men (23.01%). Similar findings were observed where men accounted for approximately two-thirds of all cases; however, women experienced a greater increase in NAFLD on a global level (50).

### Strengths and limitations

A strength of this study is the fact that it is one of the most updated meta-analyses revealing the status of MAFLD in SSA, with prevalence data that represent the general adult population. The included studies had high to moderate quality.

A limitation of this study is the high heterogeneity among studies. This could be due to the populations and areas used in the included studies and the inclusion of studies deemed of lower quality, which was all that was available. Another limitation is the possible selection bias in some of the analyses due to the small number of published studies, as seen in the funnel plot. If the MAFLD in the individuals included was diagnosed by ultrasound, which can have limited sensitivity, the prevalence of MAFLD may have been underestimated. The authors also understand that there are people with BMI <25 kg/m^2^ who have MAFLD. This was not included in the meta-analysis as it was a missing finding in the studies.

## Conclusions

In conclusion, MAFLD is a common liver disease affecting all of the three regions in SSA at a prevalence of 29.21%. There are regional variations, but also variations in ethnic groups, gender, and other comorbidities that are risk factors. These risk factors (i.e., diabetes, HTN, and obesity) highlight the significance of having metabolic control measures in place, including policies such as screening for patients with cardiometabolic risks, health education such as a change in lifestyle and increased awareness, and diagnosis.

## Data Availability

The original contributions presented in the study are included in the article/**Supplementary Material**. Further inquiries can be directed to the corresponding author.
