# Supplementary material for: Prevalence and risk factors of metabolic-associated fatty liver disease in sub-Saharan Africa: a systematic review and meta-analysis
Source: Front Gastroenterol (Lausanne). 2025 Apr 1;4:1506032. doi: 10.3389/fgstr.2025.1506032 (PMC12952399; doi:10.3389/fgstr.2025.1506032)
Supplement: Supplementary file 1 [file SupplementaryFile1.docx]

Supplementary table 1

Study characteristics.

| s/n | Author | Year of publication | Type of study | Country | Characteristics of participants | Sample size | MAFLD | Diagnosis | Type of imaging | Histological analysis | Fibrosis score |
| --- | --- | --- | --- | --- | --- | --- | --- | --- | --- | --- | --- |
| 1 | Lesi | 2009 | cross sectional | Nigeria | PLHIV | 113 | 15 | steatosis | ultrasonography | N/A | N/A |
| 2 | Kruger | 2010 | cross sectional | South Africa | overweight and obese adults | 233 | 111 | NAFLD | ultrasonography | liver biopsies | advanced fibrosis (grade 3 and 4) |
|  |  |  |  |  |  |  |  |  |  |  | no/mild fibrosis (grade 0, 1 and 2) |
| 3 | Onyekwere | 2011 | cross sectional | Nigeria | adults attending endocrine clinic | 150 | 13 | steatosis | ultrasonography | N/A | N/A |
| 4 | Elkhader | 2013 | cross sectional | Sudan | adults | 500 | 55 | NAFLD | ultrasonography | N/A | N/A |
| 5 | Almobarak | 2014 | cross sectional | Sudan | adults | 100 | 20 | NAFLD | ultrasonography | N/A | N/A |
| 6 | Almobarak | 2015 | cross sectional | Sudan | diabetic | 167 | 84 | NAFLD | ultrasonography | N/A | N/A |
| 7 | Hoffman | 2015 | retrospective | South Africa | PLHIV | 108 | 30 | steatosis | N/A | liver biopsies | N/A |
| 8 | Olusanya | 2016 | case control | Nigeria | cases diabetic | 168 | 28 | NAFLD | ultrasonography | N/A | N/A |
|  |  |  |  |  | controls non diabetic | 152 |  |  |  |  |  |
| 9 | Afolabi | 2017 | cross sectional | Nigeria | diabetic | 80 | 55 | NAFLD | ultrasonography | N/A | Grade 0: Homogenous liver parenchyma with echogenicity equal to or slightly greater than that of the renal cortex |
|  |  |  |  |  |  |  |  |  |  |  | Grade 1: Increased echogenicity of the liver with normal visualization of the diaphragm and the intrahepatic vessel borders |
|  |  |  |  |  |  |  |  |  |  |  | Grade 2: Increased echogenicity of the liver with obscuration of the walls of the portal vein branches |
|  |  |  |  |  |  |  |  |  |  |  | Grade 3: Increased echogenicity of the liver with obscuration of the diaphragmatic outline |
| 10 | Karim | 2018 | cross sectional | Kenya | adults | 246 | 33 | steatosis | Abdomina CT scan | N/A | N/A |
| 11 | Zawdie | 2018 | cross sectional | Ethiopia | diabetic | 96 | 70 | NAFLD | ultrasonography | N/A | N/A |
| 12 | Adam | 2019 | cross sectional | Sudan | adults | 292 | 43 | NAFLD | Abdomina CT scan | N/A | N/A |
| 13 | Odenigbo | 2020 | cross sectional | Nigeria | adults | 102 | 32 | NAFLD | ultrasonography | N/A | N/A |
| 14 | Setroame | 2020 | cross sectional | Ghana | Pre and post-menopausal women | 185 | 74 | NAFLD | N/A | N/A | N/A |
| 15 | Ssentongo | 2020 | cross sectional | Ghana | surgical patients | 97 | 54 | NAFLD | ultrasonography | N/A | N/A |
| 16 | Chihota | 2022 | cross sectional | Zambia | adults | 381 | 38 | steatosis | transient elastogrpahy | N/A | N/A |
| 17 | Bockarie | 2023 | cross sectional | Ghana | adults | 210 | 52 | NAFLD | ultrasonography | N/A | N/A |
| 18 | Dimitri | 2023 | cross sectional | Côte d’Ivoire | chronic viral hepatitis B | 83 | 40 | steatosis | Fibroscan | N/A | N/A |
| 19 | Lajeunesse-Trempe | 2023 | cross sectional | Kenya | adults | 640 | 94 | NAFLD | ultrasonography | N/A | N/A |
| 20 | Wiafe | 2023 | cross sectional | Ghana | diabetic | 218 | 112 | NAFLD | transient elastogrpahy | N/A | A stiffness of >7.0 kPa refers to increasing fibrosis |
|  |  |  |  |  |  |  |  |  |  |  | >7.9 kPa to 8.7 kPa consistent with histologic F2 |
|  |  |  |  |  |  |  |  |  |  |  | 8.8 to <11.7 kPa consistent with F3 fibrosis |
|  |  |  |  |  |  |  |  |  |  |  | ≥11.7 kPa consistent with advanced F4 fibrosis |
| 21 | Enriquez | 2024 | cross sectional | Uganda | adults | 1463 | 178 | MASLD | N/A | N/A | N/A |
| 22 | Kilonzo | 2024 | cross sectional | Tanzania | overweight and obese adults | 181 | 55 | NAFLD | ultrasonography | N/A | N/A |

Supplementary table 2

**Risk of bias assessment of individual studies**

| Author | standardized methods for confirming diagnosis | large enough sample size | multicenter study | appropriate statistical methods that report outcomes | Account for confounders | clear methodology of selection of participant | Population representation |
| --- | --- | --- | --- | --- | --- | --- | --- |
| Lesi |  |  |  |  |  |  |  |
| Kruger |  |  |  |  |  |  |  |
| Onyekwere |  |  |  |  |  |  |  |
| Elkhade |  |  |  |  |  |  |  |
| Almobarak |  |  |  |  |  |  |  |
| Almobarak |  |  |  |  |  |  |  |
| Hoffman |  |  |  |  |  |  |  |
| Olusanya |  |  |  |  |  |  |  |
| Afolabi |  |  |  |  |  |  |  |
| Karim |  |  |  |  |  |  |  |
| Zawdie |  |  |  |  |  |  |  |
| Adam |  |  |  |  |  |  |  |
| Odenigbo |  |  |  |  |  |  |  |
| Setroame |  |  |  |  |  |  |  |
| Ssentongo |  |  |  |  |  |  |  |
| Chihota |  |  |  |  |  |  |  |
| Bockarie |  |  |  |  |  |  |  |
| Dimitri |  |  |  |  |  |  |  |
| Lajeunesse-Trempe |  |  |  |  |  |  |  |
| Wiafe |  |  |  |  |  |  |  |
| Enriquez |  |  |  |  |  |  |  |
| Kilonzo |  |  |  |  |  |  |  |

Green cells -low risk

Grey cells – unclear risk

Red cells – high risk
